# Unconventional Spectral Gaps Induced by Charge Density Waves in the Weyl Semimetal (TaSe_4_)_2_I

**DOI:** 10.1021/acs.nanolett.4c02701

**Published:** 2024-07-08

**Authors:** Meng-Kai Lin, Joseph Andrew Hlevyack, Chengxi Zhao, Pavel Dudin, José Avila, Sung-Kwan Mo, Cheng-Maw Cheng, Peter Abbamonte, Daniel P. Shoemaker, Tai-Chang Chiang

**Affiliations:** † Department of Physics, National Central University, Taoyuan 32001, Taiwan; ‡ Department of Physics, University of Illinois at Urbana−Champaign, Urbana, Illinois 61801, United States; § Department of Materials Science and Engineering, University of Illinois at Urbana−Champaign, Urbana, Illinois 61801, United States; ∥ Synchrotron SOLEIL and Universite Paris-Saclay, L’Orme des Merisiers, BP48, 91190 Saint-Aubin, France; ⊥ Advanced Light Source, Lawrence Berkeley National Laboratory, Berkeley, California 94720, United States; # National Synchrotron Radiation Research Center, Hsinchu 30076, Taiwan

**Keywords:** (TaSe_4_)_2_I, band structure, charge density wave, Weyl semimetal, spectral gap

## Abstract

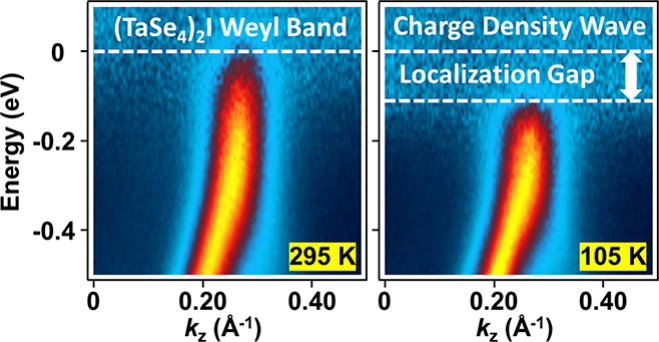

Coupling Weyl quasiparticles and charge density waves (CDWs) can lead to fascinating band renormalization and many-body effects beyond band folding and Peierls gaps. For the quasi-one-dimensional chiral compound (TaSe_4_)_2_I with an incommensurate CDW transition at *T*_C_ = 263 K, photoemission mappings thus far are intriguing due to suppressed emission near the Fermi level. Models for this unconventional behavior include axion insulator phases, correlation pseudogaps, polaron subbands, bipolaron bound states, etc. Our photoemission measurements show sharp quasiparticle bands crossing the Fermi level at *T* > *T*_C_, but for *T* < *T*_C_, these bands retain their dispersions with no Peierls or axion gaps at the Weyl points. Instead, occupied band edges recede from the Fermi level, opening a spectral gap. Our results confirm localization of quasiparticles (holes created by photoemission) is the key physics, which suppresses spectral weights over an energy window governed by incommensurate modulation and inherent phase defects of CDW.

(TaSe_4_)_2_I, with a noncentrosymmetric chiral structure in the normal phase, is a rare candidate for spontaneous transformation into an axion state of matter of interest to the particle physics and field theory communities; its CDW transition is potentially a symmetry breaking mechanism for the transformation.^1−7^ The atomic lattice of (TaSe_4_)_2_I in the normal phase, illustrated in Figure 1a, consists of TaSe_4_ molecular chains interspersed with I^–^ ionic chains. The quasi-one-dimensional structure allows easy cleavage along the (110) planes into needles along the chain direction.^4−20^ The Brillouin zone is shown in Figure 1b, where the pink rectangle represents the bulk projected Brillouin zone onto the (110) plane. Figure 1c displays the theoretical band structure of (TaSe_4_)_2_I in the normal phase; for direct comparison with experiment, the Fermi level is adjusted to account for defect-induced carrier doping.^4,7,12^ The calculated band structure along the chain direction ΓZ (Figure 1c) consists of two strongly dispersive bands A and B derived from the Ta 5*d* states, which cut through the Fermi level to form a metallic electronic structure.^12−15^ These two bands are linear near the Fermi level and cross each other to form a Weyl point W at about −0.4 eV as schematically indicated in Figure 1d. Each of the two bands is actually a spin doublet with a very small experimentally unresolvable energy splitting arising from spin–orbit coupling. Thus, there are four unresolved Weyl points at the crossing points of the two doublet bands.^5,19^ For simplicity, we will ignore the small differences and refer to the average of the four crossing points as the Weyl point arising from the crossing of bands A and B. A recent theory suggests that the Weyl points of opposite chirality can couple by nesting via a CDW lattice modulation, leading to a topological CDW gap at the Weyl point (Figure 1e). The resulting axion insulator phase can host anomalous electronic and nonlinear magneto-transport properties.^5,6^ While this theory is conceptually appealing and elegant, relevant experimental evidence based on transport measurements has been called into question.^8^ A further issue, at the most basic level, is that the experimental CDW wave vectors (±0.045, ±0.045, ±0.085)^5,21,22^ are inconsistent with the Peierls or axion nesting condition.^8^

**Figure 1:**
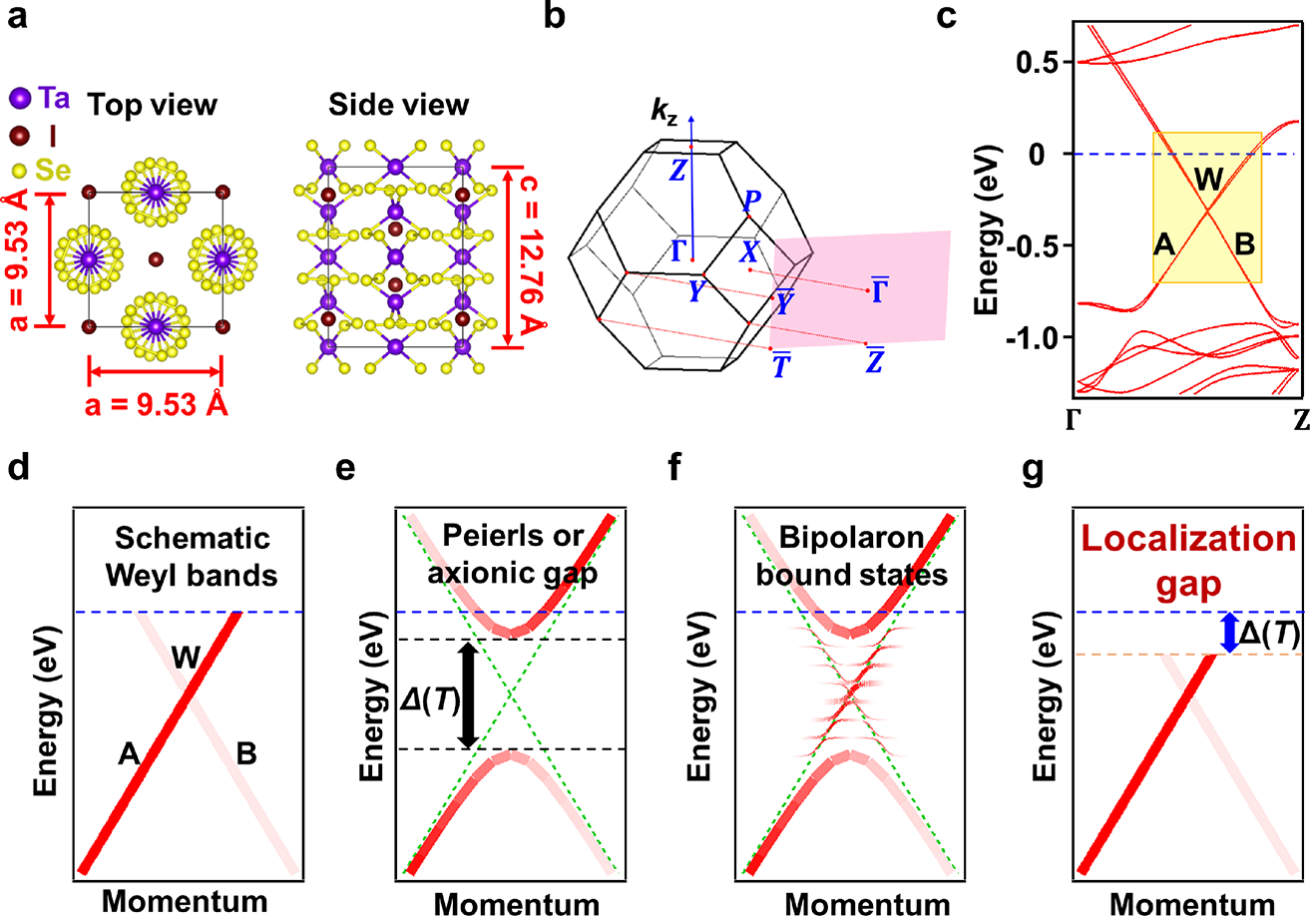
Crystal structure and band structure of (TaSe_4_)_2_I. (a) Schematic atomic structure. (b) Brillouin zone and the surface projection. (c) Calculated band structure along the chain direction ΓZ in the normal phase. The two bands labeled A and B cross the Fermi level, and the system is metallic. The same two bands cross each other to form a Weyl point labeled W. (d) Schematic representation of the Weyl band structure near the Fermi level. (e) Schematic band structure assuming a Peierls or axion anticrossing gap opening at the Weyl point. Note that for a small gap opening, the system should remain metallic. (f) Another model prediction involving bipolaron bound states within the gap. (g) Key finding of our work is a temperature-dependent spectral gap Δ(*T*) in the bands just below the Fermi level.

Angle-resolved photoemission spectroscopy (ARPES) would be the best method to clarify the nature of the CDW by band mapping to reveal the presumed nesting or axion gap opening at the Weyl point (Figure 1e) with a characteristic dependence on temperature. Our ARPES results of band mapping along the chain direction ΓZ (Figure 2a, defined as the *z* direction) at *T* = 295 K in the normal phase are consistent with earlier experimental findings. Seen in the data on the +*k*_*z*_ side are an intense band A, a much weaker band B, and a bundle of other bands below −1 eV, in good accord with theory for the normal phase (Figure 1c).^5,12^ The ARPES results on the −*k*_*z*_ side are a mirror copy as expected but with different intensities. Data taken at *T* = 105 K deep in the CDW phase (Figure 2b) show the same band dispersions with no evidence for a gap at the Weyl point. A puzzling observation, as has been reported before,^5,7,9,12,15^ is that bands A and B are very weak above the Weyl point and not obvious in the raw data (Figures 2a and 2b), and it is unclear if the system is actually metallic as suggested by the theoretical band structure. This lack of intensity has been attributed to polaronic effects involving multiphonon sidebands in accordance with the Franck–Condon principle^15,23^ or formation of bipolaron states as a set of in-gap flat bands in the ground state.^9^ However, these predicted polaronic features are absent or unclear experimentally under static conditions.

**Figure 2:**
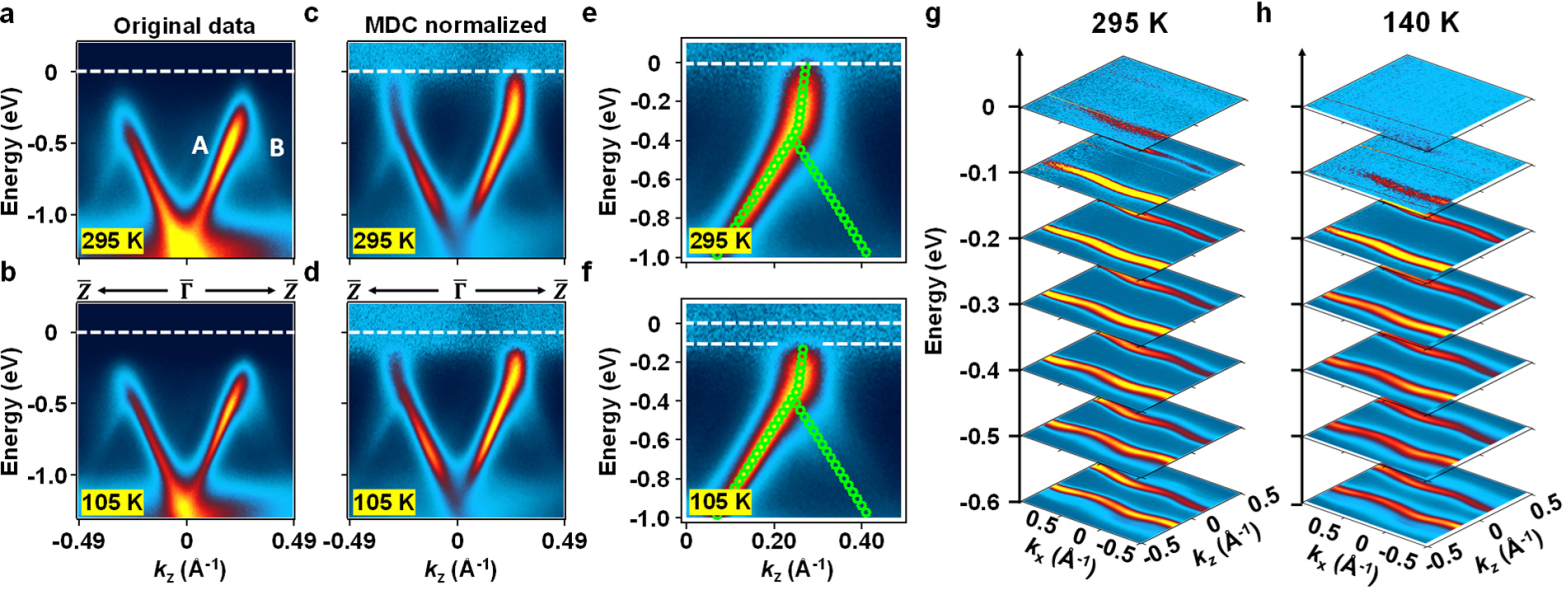
ARPES band mapping results. (a) ARPES map along *k*_*z*_ (the chain direction ΓZ) at *T* = 295 K taken with 25 eV photons. (b) Corresponding map at *T* = 105 K. (c) Same as (a) but with MDC normalization of the intensity to reveal the bands above the Weyl point. (d) Same as (b) but with MDC normalization of the intensity. A spectral gap is evident just below the Fermi level. (e) A zoomed-in view of (c) on the +*k*_*z*_ side with fitting of the bands (green circles) to extract the position of the Weyl point at *E*_W_ = −0.384 eV and a bosonic kink at *E*_K_ = −0.343 eV. (f) Similar to (e) but for *T* = 105 K. The Weyl point and the kink remain the same, but a spectral gap opens below the Fermi level. (g) Constant energy cuts of the ARPES maps at *T* = 295 K taken with 51 eV photons. (h) Similar to (g) but for *T* = 140 K.

The apparent missing upper branches of the bands in the normal phase are made visible in Figure 2c, which is based on the same data in Figure 2a but with the intensity renormalized such that the integrated intensity for each horizontal data slice (momentum distribution curve or MDC) as a function of *E* is set to be the same. This MDC normalization compresses the large intensity variations along the energy axis, thus facilitating the visualization of the entire dispersion relation. Evidently, the bands for the normal phase at *T* = 295 K do reach the Fermi level, confirming a metallic state. The green circles in Figure 2e, a zoomed-in view of Figure 2c, indicate the band dispersion relations extracted from peak fitting. The two bands A and B are quite linear below about −0.4 eV, and the Weyl point as the point of band intersection is determined to be *E*_W_ = −0.384 eV. Interestingly, the portion of band A above the Weyl point, now visible, does not follow a linear extrapolation from the same band below the Weyl point. Fitting (green circles) reveals a kink in the otherwise linear band A at *E*_K_ = −0.343 eV, which is slightly above the Weyl point. Such “bosonic kinks” have been observed in many materials and can be attributed to electron–phonon coupling not included in standard band calculations.^24−28^ The detailed shapes of the kinks have attracted much interest.^24,26,29−31^ For the present case, we do not have a detailed theory but hope that our results will spur strong theoretical interest. Figures 2d and 2f present corresponding MDC-normalized results for the CDW phase at *T* = 105 K. A key finding is that a small spectral gap appears just below the Fermi level such that the upper portions of the bands become simply missing. The band shapes including the Weyl points and the kinks remain otherwise intact. The missing spectral weight near the Fermi level in the CDW phase is further confirmed by comparing the constant-energy ARPES maps as a function of the in-plane momentum in Figures 2g and 2h measured at *T* = 295 and 140 K, respectively. The Fermi contours (*E* = 0) at *T* = 295 K are sharp and appear almost straight along *k*_*z*_ (parallel to the chains) with slight wiggles along *k*_*x*_ (perpendicular to the chains in the surface plane), in agreement with the quasi-one-dimensional nature of the system.^4−22^ These Fermi contours disappear at *T* = 140 K.

A zoomed-in view of the MDC-normalized spectral function on the +*k*_*z*_ side as a function of temperature (Figure 3b) confirms no Periels or axion anticrossing gaps at the Weyl point. The visible portion of the dispersion relation including the kink position at *E*_K_ = −0.343 eV remains the same over the entire temperature range of 105–295 K. The main effect of the temperature dependence is that the top edge of the band gradually recedes from the Fermi level as *T* is lowered below *T*_C_. This special gap opening is not apparent in the original data without MDC normalization (Figure 3a). The very weak ARPES intensity near the Fermi level makes it difficult to discern the gap opening as noted in prior studies. MDC normalization and high data statistics are key to uncovering the gap behavior.

**Figure 3:**
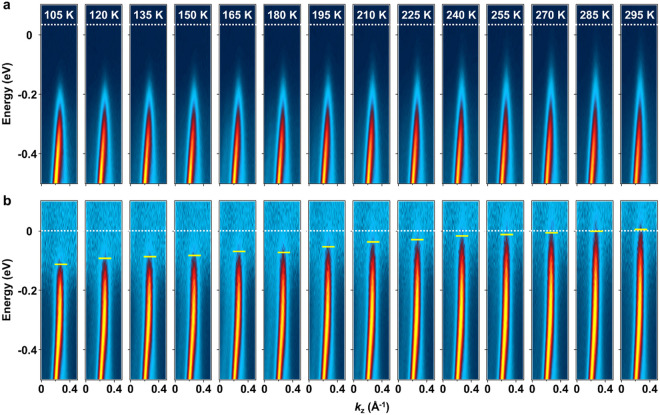
Detailed view of the bands near the Fermi level as a function of temperature. (a) ARPES maps on the +*k*_*z*_ side at various temperatures. (b) The same results with MDC normalization of the intensity. The short horizontal lines indicate the band edges, which move below the Fermi level as *T* lowers below *T*_C_.

To quantify the spectral gap opening, each MDC at energy *E* in the original data set (without MDC normalization to avoid possible data distortion) is analyzed by fitting to extract the ARPES intensity from the band at each temperature. The results (Figure 4a) show the rapid intensity decrease near the Fermi level. The same results plotted using a logarithmic intensity scale (Figure 4b) better illustrate the recession of the leading edge of the band from the Fermi level represented by a vertical line at *E*_F_ = 0. The curves for *T* > *T*_C_ cut through *E*_F_; the average of the intensities at *E*_F_, indicated by a horizontal line in Figure 4b, is taken to indicate the band edge intensity for the normal phase at the Fermi level. The cuts of the curves for *T* < *T*_C_ with the horizontal line are taken to represent the corresponding receding band edges. The edge positions so determined are indicated by short horizontal lines in Figure 3b, which agree well with a visual inspection of the data. Figure 4c shows the spectral gap, defined as the energy difference between the Fermi level and the edge position, as a function of *T*. The curve in Figure 4c is a fit using the empirical gap equation
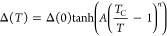
1where *T*_C_ = 263 K.^5−22^ The parameters extracted from the fitting include the zero-temperature gap Δ(0)= 0.108 ± 0.002 eV, the bowing parameter *A* = 1.29 ± 0.05, and the critical exponent *n* = 0.85 ± 0.02. For comparison, *n* = 1/2 for a mean-field second-order transition. Most simple superconductors are well-described by the BCS theory, which is based on a mean-field treatment, and eq 1 with *n* = 1/2 agrees well with the experimental superconducting gaps for those systems.^32^ However, a mean-field theory ignores fluctuation effects and is not expected to hold for quasi-one-dimensional systems such as (TaSe_4_)_2_I. Indeed, the value of *n* in our case deviates significantly from the mean-field value.

**Figure 4:**
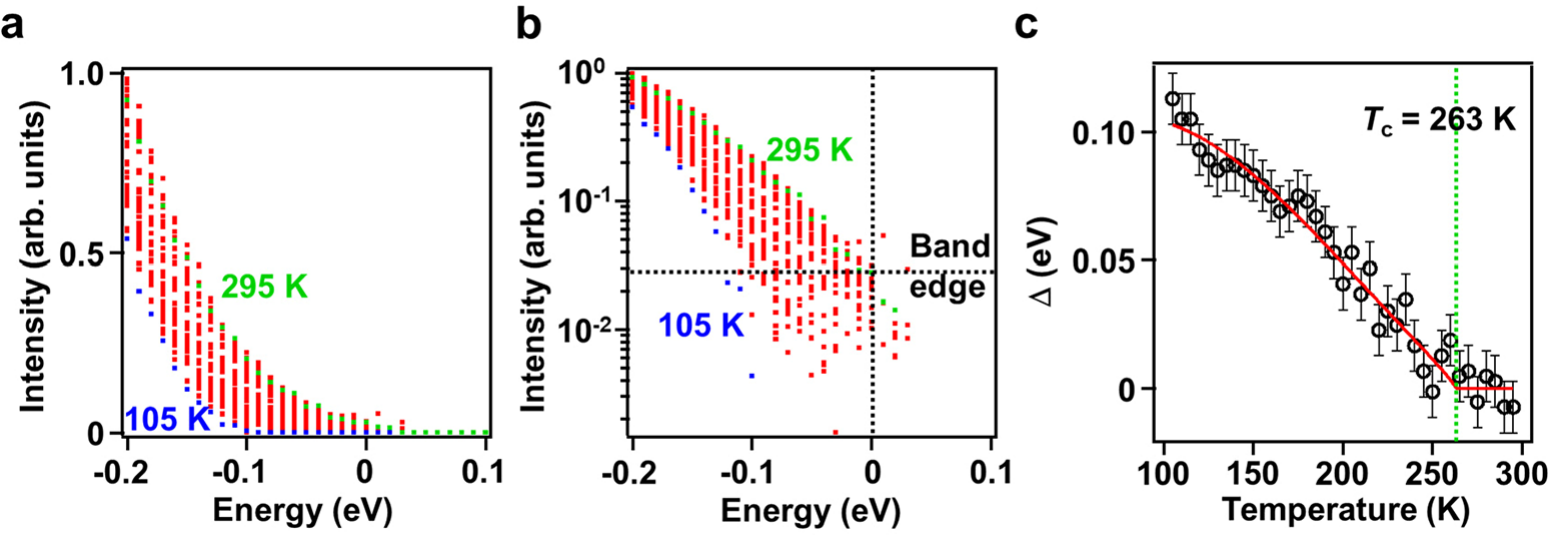
Determination of the spectral gap. (a) Intensity of the band seen in Figure 3a extracted from fitting the ARPES peak as a function of *E* for various temperatures. (b) The same results plotted using a logarithmic intensity scale. The vertical line indicates the Fermi level, and the horizontal line indicates the band edge intensity for the normal phase at the Fermi level. The cuts of the curves for *T* < *T*_C_ with the horizontal line are taken to be the corresponding band edges. The energy difference between the edge and the Fermi level is the spectral gap. (c) The extracted spectral gap is plotted as a function of temperature with error bars. The curve is a fit using a model function described in the text.

The behavior of the spectral gap (Figure 3b) indicates unconventional CDW physics. The underlying mechanism cannot be Peierls or axion anticrossing coupling. Neither can it arise from dynamic scattering from phonons, amplitudons, or phasons because such effects should diminish at low temperatures. In the limit of *T* → 0, the phase space for quasiparticle scattering by dynamic lattice modes vanishes. However, because the phase of the incommensurate CDW is not tied to the lattice, there can be numerous topological defects in the form of phase slips at phase domain boundaries. These static phase defects create potential barriers, which can trap the quasiparticles (holes created by ARPES) at low excitation energies and render them localized. In this picture, the gap Δ(0) can be identified as a measure of the strength of the potential barriers for quasiparticle trapping at *T* = 0. The portions of the dispersion relations within Δ(0) from the Fermi level should disappear with the spectral weight scattered diffusively in momentum space because a localized state has no momentum dependence. Quasiparticles with excitation energies greater than Δ(0) remain delocalized and retain their dispersion relations in the normal phase. As *T* increases from 0 toward *T*_C_, the amplitude of the CDW diminishes, and the potential barriers associated with the phase defects also diminish, leading to a reduced spectral gap. The gap in eq 1 is thus related to the CDW amplitude as the order parameter of the transition. Our ARPES data were limited to *T* greater than ∼105 K; the samples became insulating and showed charging effects at lower temperatures.

A related issue is the rapid reduction of the ARPES intensity above the Weyl point as seen in Figures 2a and 2b in addition to the gapping effects mentioned above. The original data reveal a rapid reduction of the ARPES intensity beginning at about −0.38 eV, reaching 50% at around −0.28 eV for the entire temperature range (Supplementary Figure 3). This sharp intensity drop-off correlates well with the kink energy at *E*_K_ = −0.343 eV. A straightforward interpretation is that the strong coupling of the quasiparticles to the lattice bosonic modes responsible for the kink leads to diffuse scattering and the observed attenuation of the coherent ARPES spectral weight.

Our results of ARPES mapping of the bands show that the conventional pictures for the CDW in (TaSe_4_)_2_I based on Peierls or axion gap opening (Figure 1e) and bipolaronic bound states (Figure 1f) do not apply. Rather, it is the emergence of a *T*-dependent spectral gap Δ(*T*) (Figure 1g), rendered visible in our study with a high level of data statistics covering a wide range of ARPES intensities. The Weyl point, its nearby band dispersion, and the prominent band kink just above the Weyl point have no direct bearing on the CDW. Our observations suggest quasiparticle (hole) localization caused by incommensurate CDW phase defects is the underlying physics for the unconventional band structure evolution. The ground state in the *T* → 0 limit is thus a phase-defect-induced insulating phase.

## Methods

Single crystals of (TaSe_4_)_2_I were prepared by chemical vapor transport.^20^ Stoichiometric quantifies of high purity Ta wire, Se powder, and I shot were loaded into a fused silica tube, which was sealed under vacuum and heated with a source temperature of 600 °C and sink temperature of 500 °C for 10 days. The crystals were characterized by resistivity measurements and by X-ray diffraction on a Bruker D8 ADVANCE diffractometer with Mo Kα radiation; the measured temperature dependence confirmed a transition temperature of *T*_C_ = 263 K.^33,34^ ARPES measurements were performed using various photon energies on samples freshly cleaved in situ at beamline 21B, Taiwan Light Source (TLS), beamline 10.0.1.1, Advanced Light Source (ALS), and beamline ANTARES, Synchrotron SOLEIL. The measuring time for each sample after cleavage was limited to a day, during which no noticeable changes in ARPES results were observed.

## Supporting Information Available

(1) (TaSe_4_)_2_I single crystals and cleavage; (2) sample characterization; (3) temperature-dependent ARPES intensity of band A in (TaSe_4_)_2_I; (4) retention of spectral shape below the spectral gap; (5) spatial mapping and polarization dependence of ARPES maps; (6) relationship between the gap and the transition temperature; and (7) peak widths of MDC measurements (PDF)

## Abbreviations

CDW: charge density wave
ARPES: angle-resolved photoemission spectroscopy
MDC: momentum distribution curve
