# Supplementary material for: Unconventional Spectral Gaps Induced by Charge Density Waves in the Weyl Semimetal (TaSe4)2I
Source: Nano Lett. 2024 Jul 8;24(28):8778–83. doi: 10.1021/acs.nanolett.4c02701 (PMC11261618; doi:10.1021/acs.nanolett.4c02701)
Supplement: Supplementary file 1 — nl4c02701_si_001.pdf [file nl4c02701_si_001.pdf]

Supporting Information for "Unconventional Spectral Gaps Induced by Charge Density Waves in the Weyl Semimetal $(TaSe_4)_2I$"

Meng-Kai Lin[1,*], Joseph Andrew Hlevyack[2], Chengxi Zhao[3], Pavel Dudin[4], José Avila[4], Sung-Kwan Mo[5], Cheng-Maw Cheng[6], Peter Abbamonte[2], Daniel P. Shoemaker[3], and Tai-Chang Chiang[2,*]

[1]Department of Physics, National Central University, Taoyuan 32001, Taiwan

[2]Department of Physics, University of Illinois at Urbana-Champaign, Urbana, Illinois 61801, USA

[3]Department of Materials Science and Engineering, University of Illinois at Urbana-Champaign, Urbana, Illinois 61801, USA

[4]Synchrotron SOLEIL and Universite Paris-Saclay, L'Orme des Merisiers, BP48, 91190 Saint-Aubin, France.

[5]Advanced Light Source, Lawrence Berkeley National Laboratory, Berkeley, California 94720, USA

[6]National Synchrotron Radiation Research Center, Hsinchu 30076, Taiwan

Corresponding Authors

[*]Meng-Kai Lin, Email: mklin@office365.ncu.edu.tw

[*]Tai-Chang Chiang, Email: tcchiang@illinois.edu

$(TaSe_4)_2I$ single crystals and cleavage

Figure S1a shows as-grown single crystals of $(TaSe_4)_2I$ stored under inert gas in a glass container. The crystals, with a quasi-one-dimensional atomic structure, have needle-like shapes and tend to break along the (110) surfaces after cleavage. Figure S1b shows a sample after cleavage. The surface appears corrugated, which, however, does not affect ARPES band mapping along the ΓZ chain direction. Each sample for ARPES measurements is attached to a copper plate using silver epoxy to ensure good thermal conductivity.

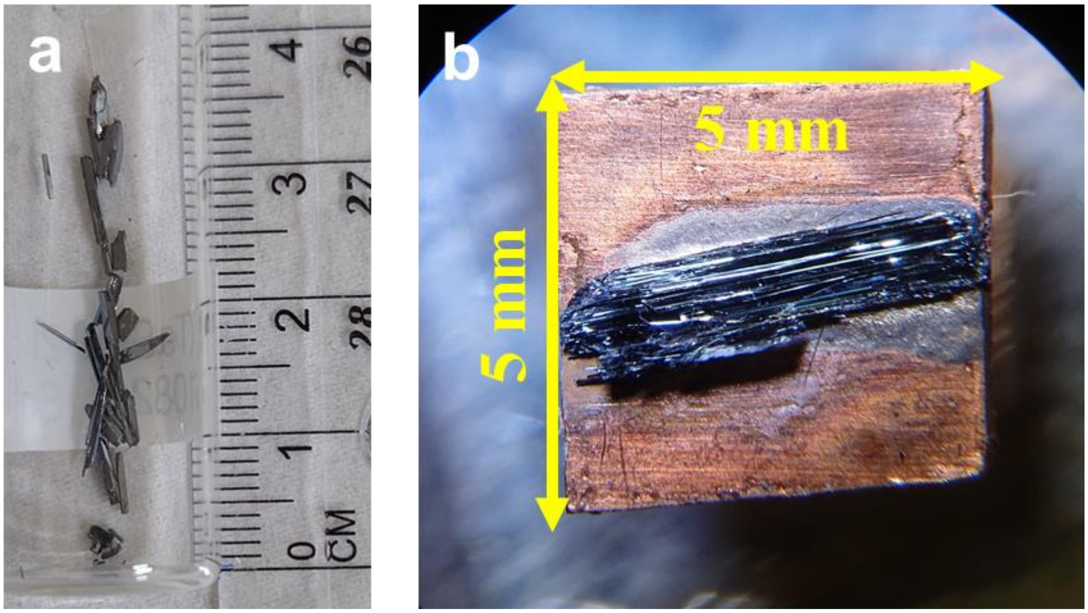

Figure S1 a As-grown single crystals of $(TaSe_4)_2I$. **b** A $(TaSe_4)_2I$ crystal after cleavage.

Sample characterization

The $(TaSe_4)_2I$ material was characterized by x-ray diffraction and resistivity measurements. As shown in Fig. S2a, CDW satellite peaks emerge as the sample temperature is lowered below T_C. Plotted in Fig. S2b is the intensity of the satellite peak as a function of temperature. The results

indicate a transition temperature of T_C = 263 K. The sample resistivity measured along the chain direction as a function of temperature, shown in Fig. S2c, shows a subtle change in slope around T_C. Upon taking the logarithmic derivative of the data, a sharp peak is seen at 263 K. These results confirm the transition temperature at T_C = 263 K, in agreement with the accepted value in the literature.

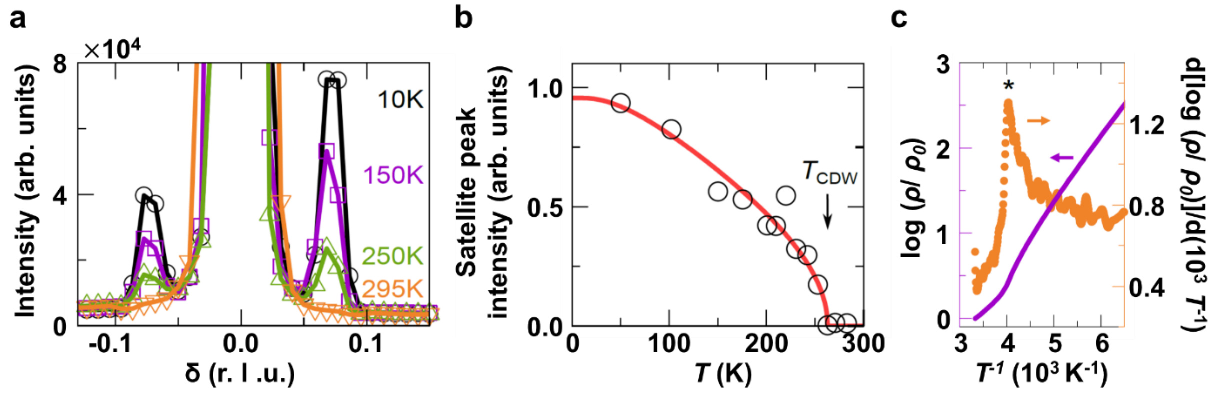

Figure S2 Sample characterization. a Line cuts of temperature-dependent x-ray diffraction taken around the Bragg reflection at (*h*, *k*, *l*) = (-1, 3, 4). **b** Temperature-dependent intensity of the CDW satellite peaks. **c** Logarithmic resistivity (normalized by $\rho_0 = \rho$(300 K)) and its derivative as a function of 1/*T*.

Temperature-dependent ARPES intensity of band A in $(TaSe_4)_2I$

Figure S3 shows ARPES intensity of band A (Fig. 1) as a function of energy at various temperatures. The intensity for each temperature is normalized by its maximum within the selected energy range (–0.4 to 0.1 eV). The results show a rapid reduction beginning at about –0.38 eV, reaching 50% at around –0.28 eV for the entire temperature range. The intensity is extremely low at the Fermi level at E = 0.

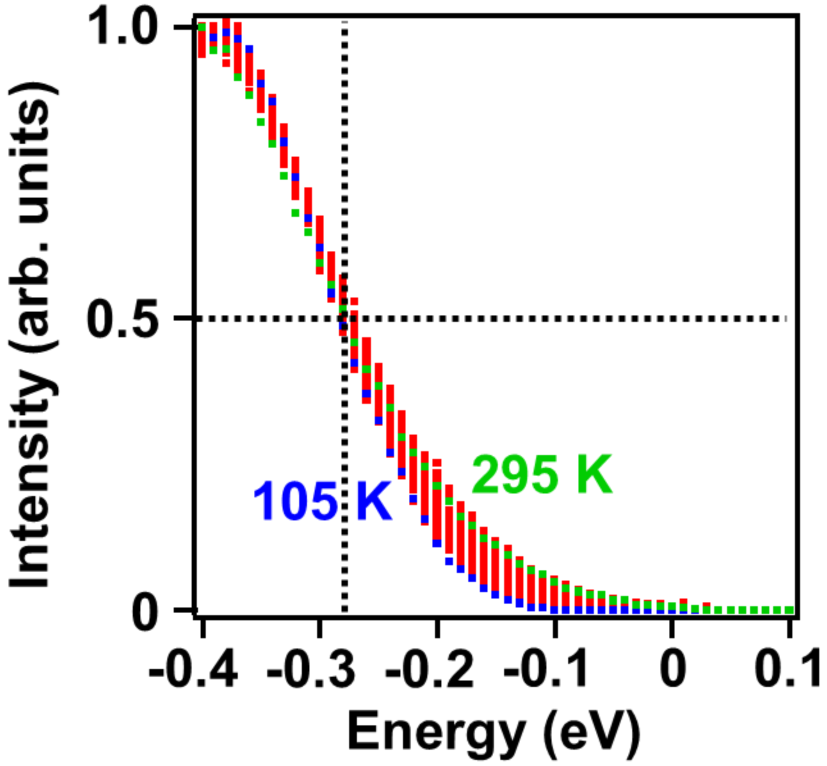

Figure S3 Temperature dependence of ARPES intensity of band A. The blue and green dotted curves represent data taken at 105 and 295 K, respectively. The red dotted curves represent data taken at temperatures in-between.

Retention of spectral shape below the spectral gap

Figure S4a shows an ARPES map taken at 105 K with 25-eV photons. Three vertical lines are drawn at k_z = 0.13, 0.19, and 0.25 Å^{-1}. Figure S4b shows energy distribution curves (EDCs) at k_z = 0.25 Å^{-1} (location of the Weyl point) as a function of temperature. The energy position and the shape of the peak at the Weyl point remains unchanged as a function of temperature with no evidence for a Peierls or axionic gap. The peak positions of band A from curve fitting are shown in Fig. S4c for the three selected k_z values over the temperature range covered in the experiment

(105 to 295 K). These peak positions do not show significant temperature dependence. Thus, the band structure remains unchanged except for the appearance of a spectral gap very close to the Fermi level.

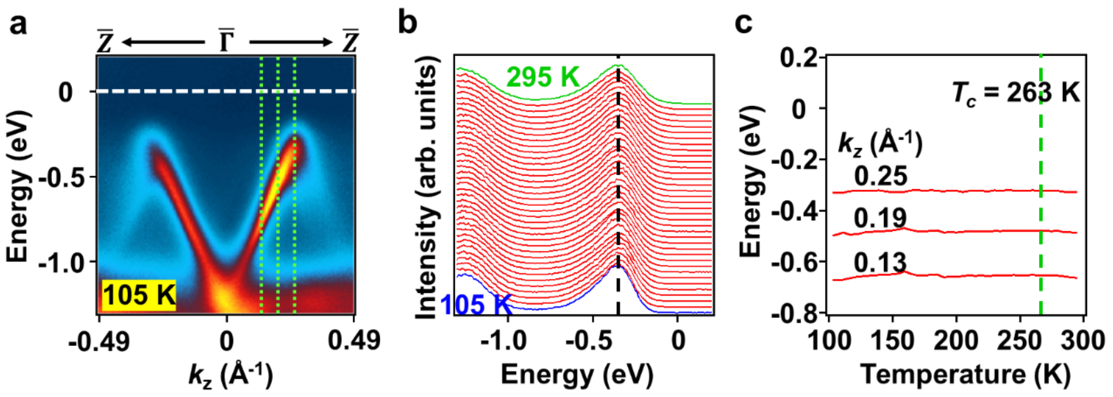

Figure S4 **a** ARPES map taken at 105 K with 25-eV photons. **b** EDCs taken at k_z = 0.25 Å^{-1} as a function of temperature. Blue, green, and red curves correspond to 105 K, 295 K, and temperatures in-between, respectively. **c** Energy positions of the band (cuts by the green dotted lines in **a**) as a function of temperature.

Spatial mapping and polarization dependence of ARPES maps

Figure S5 shows the experimental geometry for spatial mapping with four different polarization configurations (LV = linear vertical, LH = linear horizontal, CR = circular right, and CL = circular left). The incident photon energy was 51 eV. The angle between the incident beam and the sample plane was 45°, and the size of the beam focus was 3 µm. The sample was kept at 127 K during the measurements. The time for imaging over a selected area of 150 × 200 µm^2 was about one hour for each polarization configuration.

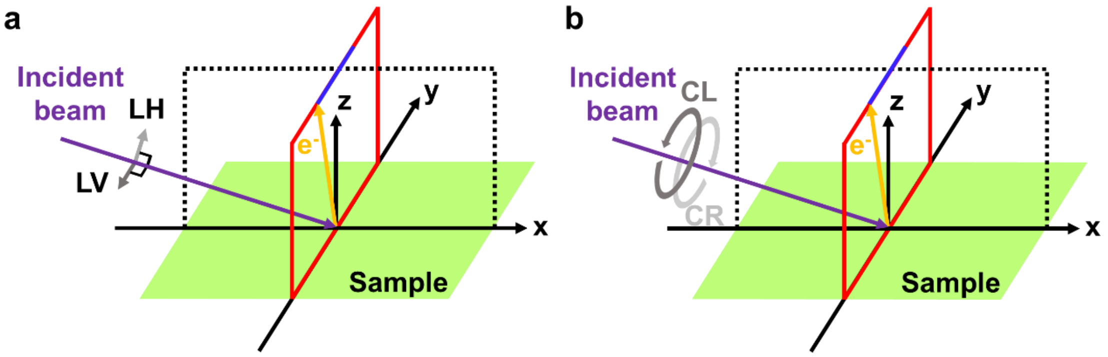

Figure S5 Polarization configuration for spatial mapping. a For linearly polarized light. **b** For circularly polarized light.

Figures S6a-S9a show spatial ARPES maps over the same 150 × 200 μm^2 area for the four different polarization configurations. The signal corresponds to ARPES intensities integrated over the energy range of 0 to –1 eV and the momentum range of 0 to 0.5 Å^{-1} (this region is indicated in Fig. S6b-S9b). There are substantial variations over the imaged area for each polarization configuration. The fractional variation $\Delta I/\bar{I}$ (root mean square deviation from the mean normalized by the mean) is 14%, 10%, 14%, and 12% for the LV, LH, CR, and CL polarization configurations, respectively. It is interesting to note that the spatial features of the four images show some correlations but are not identical. A likely reason for the complexity is surface roughness (Fig. S1). Theoretically, ARPES intensities can depend strongly on the surface orientation relative to the electric field of the incident beam[1]. For a rough surface, the polarization dependence can become quite complex. Figures S6b-S9b are ARPES results obtained by integrating over the whole scanned area of 150 × 200 μm^2.

To further explore the spatial variations, the ARPES results integrated over four randomly selected 20 × 20 μm^2 areas, indicated by red squares labeled 1-4 in Figs. S6a-S9a, are shown in

Figs. S6-S9c-f. Evidently, the observed band dispersion relations are the same for the different areas, as expected for mapping along the ΓZ chain direction, despite the intensity variations caused by surface roughness.

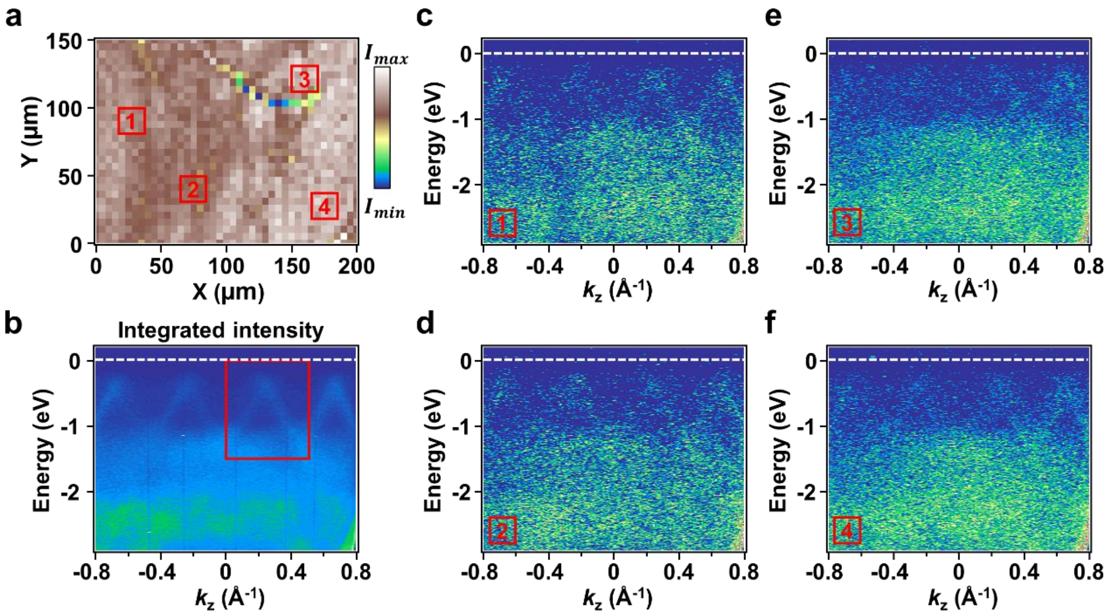

Figure S6 ARPES maps taken with linear vertically polarized light at 51 eV. a Spatial mapping over an area of 150 × 200 μm^2. **b** ARPES results integrated over the imaged area in **a**. **c-f** ARPES results taken over four randomly selected 20 × 20 μm^2 areas, labeled 1-4 in **a**.

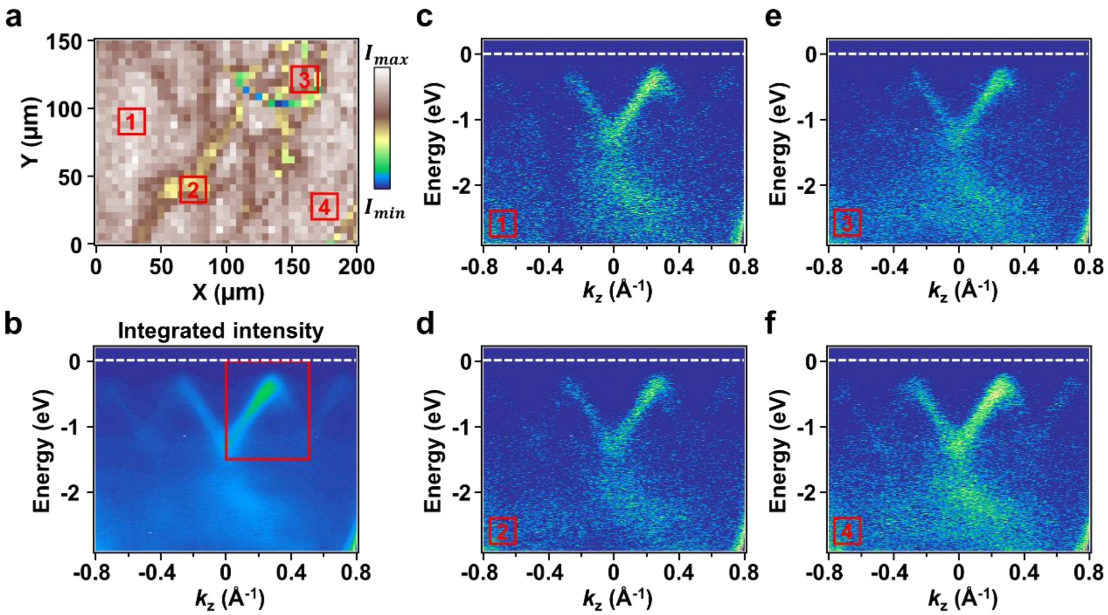

Figure S7 ARPES maps taken with linear horizontally polarized light. Same as Fig. S6 except for the different polarization.

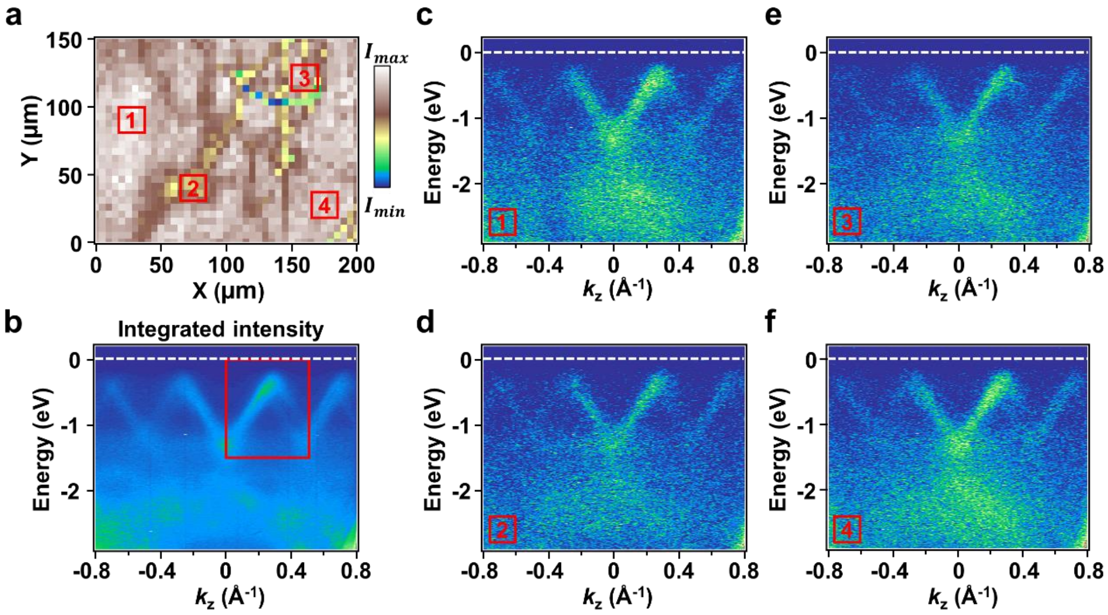

Figure S8 ARPES maps taken with right circularly polarized light. Same as Fig. S6 except for the different polarization.

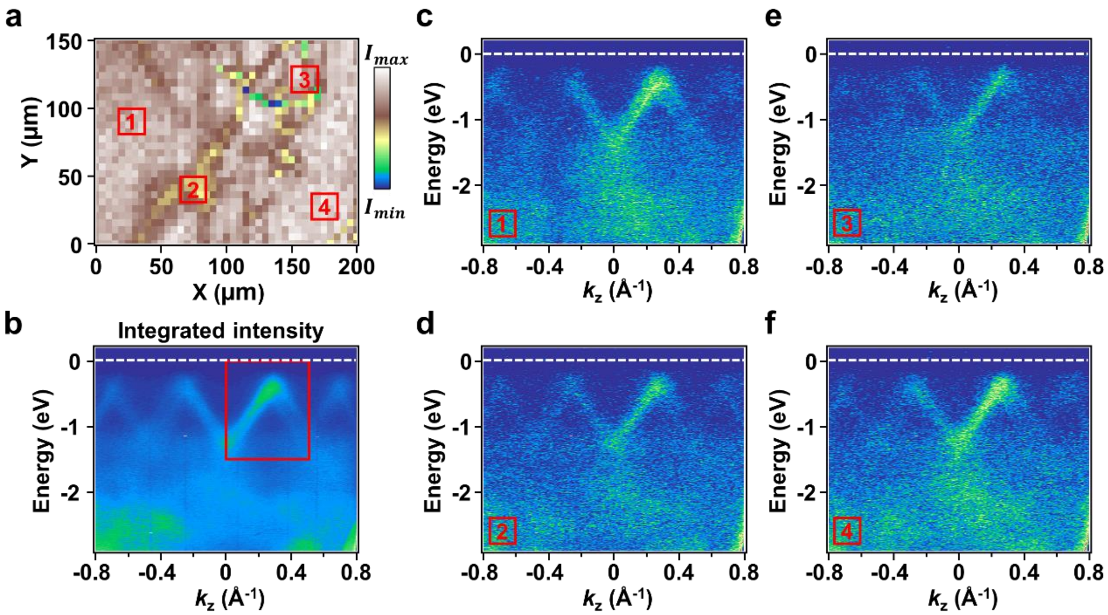

Figure S9 ARPES maps taken with left circularly polarized light. Same as Fig. S6 except for the different polarization.

Relationship between the gap and the transition temperature

The zero-temperature gap $\Delta(0)$ = 0.108 eV extracted from our data (Fig. 4) is related to the transition temperature T_C = 263 K by

$$\frac{2\Delta(0)}{k_B T_C} = 9.53.$$

For comparison, single-layer VSe_2 shows a $\left(\sqrt{7}\times\sqrt{3}\right)$ CDW transition at T_C = 220 K, below which a gap in the dispersion relation opens at the Fermi level with a clean second-order behavior[2]. With $\Delta(0)$ = 0.101 eV, the above ratio becomes

$$\frac{2\Delta(0)}{k_B T_C} = 10.6$$

which is very similar to that for $(TaSe_4)_2I$. In general, this ratio can change from system to system depending on the specifics, but it should be similar in magnitude. The reason is that $\Delta(0)$ is a measure of the energy scale for ordering, and it must be related to $k_B T_C$, a measure of the energy of thermal fluctuation that destroys the CDW order at T_C.

Superconducting and CDW transitions often compete. For superconducting transitions, a similar ratio is of interest. It is 3.53 based on the BCS theory, but the experimental values range from about 3 to 8 for most cases including high-temperature superconductors. It is interesting to note that the effective width of the Fermi-Dirac distribution function at T equals $3.53 k_B T$, where the same numerical factor 3.53 appears. More precisely, the negative derivative with respect to energy of the Fermi-Dirac distribution function, which appears in physical property calculations involving thermal broadening[3], has the shape of a symmetric peak with a full width at half maximum of $3.53 k_B T$. This is a good measure of the effective energy broadening of the charge carriers at temperature T.

Peak widths of MDC measurements

Figure S10 shows waterfall plots of MDCs at 295 and 105 K. The peak width is essentially constant (~0.07 $Å^{-1}$) independent of temperature and energy. This observation suggests that the MDC peak width is likely dominated by defect scattering along the chains. The energy broadening, which is related to momentum broadening by the group velocity, is also essentially constant. The defects may include iodine deficiency and a rough surface from cleavage.

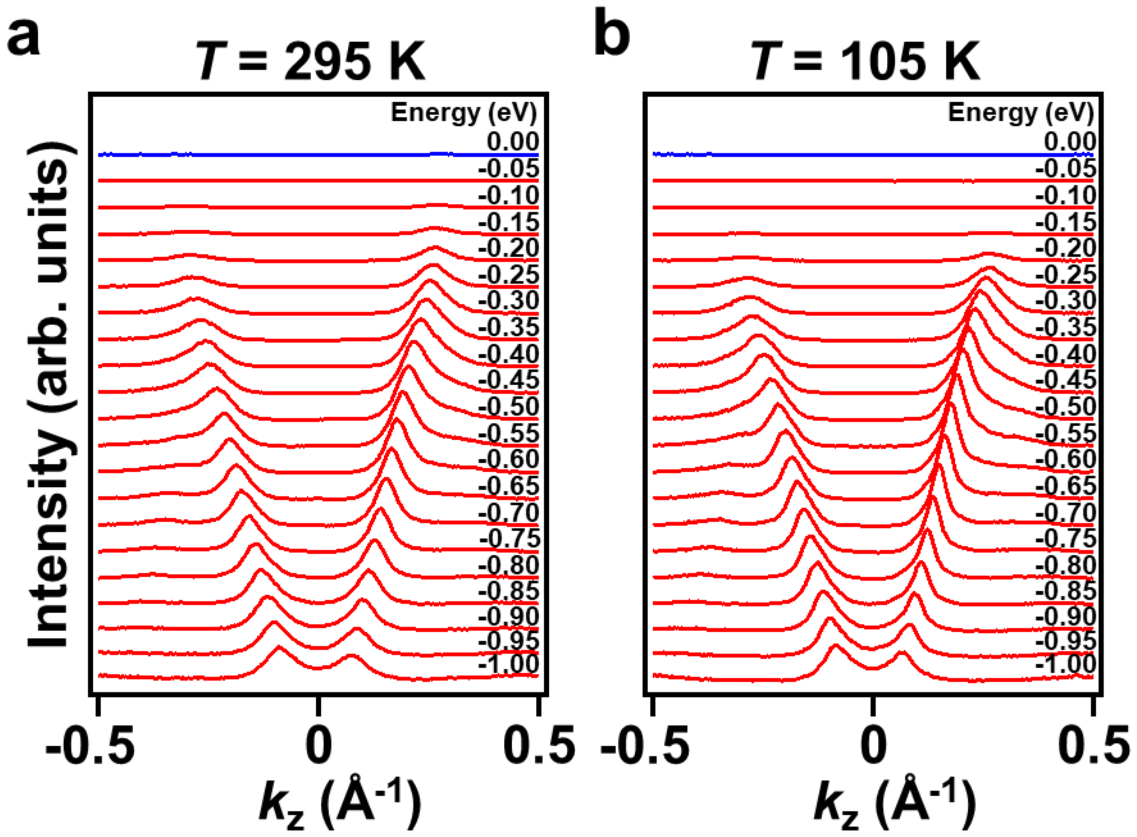

Figure S10 Waterfall plots of MDCs. a, b Momentum distribution curves taken at 295 and 105 K, respectively.

Supporting references

1. Xu, C.-Z. et al. Photoemission circular dichroism and spin polarization of the topological surface states in ultrathin Bi_2Te_3 films. *Phys. Rev. Lett.* 115, 016801 (2015).
2. Chen, P. et al. Unique gap structure and symmetry of the charge density wave in single-layer VSe_2. *Phys. Rev. Lett.* 121, 196402 (2018).
3. Grimvall, G. *The Electron-phonon Interaction in Metals* (Elsevier North-Holland, 1981).
